# Classical and Modern Approaches Used for Viral Hepatitis Diagnosis

**DOI:** 10.5812/hepatmon.17632

**Published:** 2014-04-16

**Authors:** Mohammad Heiat, Reza Ranjbar, Seyed Moayed Alavian

**Affiliations:** 1 Molecular Biology Research Center, Baqiyatallah University of Medical Sciences, Tehran, IR Iran; 2 Middle East Liver Disease Center (MELD), Tehran, IR Iran

**Keywords:** Hepatitis Viruses, Immunoassay, Biosensing Techniques

## Abstract

**Context:**

Viral hepatitis diagnosis is an important issue in the treatment procedure of this infection. Late diagnosis and delayed treatment of viral hepatitis infections can lead to irreversible liver damages and occurrence of liver cirrhosis and hepatocellular carcinoma. A variety of laboratory methods including old and new technologies are being applied to detect hepatitis viruses. Here we have tried to review, categorize, compare and illustrate the classical and modern approaches used for diagnosis of viral hepatitis.

**Evidence Acquisition:**

In order to achieve a comprehensive aspect in viral hepatitis detection methods, an extensive search using related keywords was done in major medical library and data were collected, categorized and summarized in different sections.

**Results:**

Analyzing of collected data resulted in the wrapping up the hepatitis virus detection methods in separate sections including 1) immunological methods such as enzyme immunoassay (EIA), radio-immunoassay (RIA) immuno-chromatographic assay (ICA), and immuno-chemiluminescence 2) molecular approaches including non-amplification and amplification based methods, and finally 3) advanced biosensors such as mass-sensitive, electrical, electrochemical and optical based biosensors and also new generation of detection methods.

**Conclusions:**

Detection procedures in the clinical laboratories possess a large diversity; each has their individual advantages and facilities' differences.

## 1. Context

Viral hepatitis is caused by several viruses known as hepatitis viruses. The most common types of hepatitis viruses include hepatitis A virus (HAV), B (HBC), C (HCV), D (HDV) and E (HEV). About 1.4 million people become infected by HAV around the world annually. More than 240 million people live with chronic HBV infection and about 600 thousand people die every year from acute or chronic HBV infection. About 150 million people globally have chronic HCV infection and more than 350 thousand people die every year due to HCV-related liver diseases (1). Hepatitis viruses are different in genomic type, antigenic patterns, mode of transmission, severity and chronicity, etc. (2). During recent years, some considerable efforts have been devoted toward development of various techniques to detect hepatitis virus derivative targets including: IgM and IgG for HAV, all serological markers (anti-HBc antibodies [IgM and IgG], anti-HBe antibody, HBeAg and HBsAg), and HBV DNA for HBV (3), anti-HCV antibodies and HCV RNA for HCV, anti-HDV antibodies and HDV antigen for HDV and anti-HEV antibodies and HEV RNA for HEV (4). Immunoassay methods were described as general and versatile techniques for identification of pathogenic agents (5). The most commonly and also traditionally used immunoassay techniques for detection of hepatitis viruses are RIA (6, 7) and ELISA (8). Immunochemiluminescent assay, and lateral flow immunoassay are among more advanced immunoassay techniques. Recently, molecular approaches have been converted into unavoidable implements to diagnose viral disease, with accurate and reliable results (9, 10). Widely utilized molecular methods for detection of hepatitis viruses relies on nucleic acid amplification. Signal, target and probe amplifications are described as formats of amplification based methods (11, 12). Nowadays the modern techniques for hepatitis detection are intellectual combinations between serological and molecular methods, jointed with nanotechnology, chemistry, electronics and high-tech apparatus. On the other hand, some newfound recognizer elements such as aptamers are emerging to help scientists to identify the hepatitis virus easier than past (13). In this review, it was intended to investigate the classical and modern techniques in diagnosis of hepatitis viruses and introduce the developed and advanced methods available in this field. 

## 2. Evidence Acquisition

In this review article after determination of the main concepts and statement about the problem, attempts made to gather data about the aims and aspects, from major databases including PubMed, EMBASE and Scopus from 1970 to 2013. In this regard the most relevant keywords such as hepatitis viruses, classical and novel detection techniques, immunoassay, biosensors, molecular detection methods and many more were used to construct thesaurus in different databases. Irrespective to any classification, about 120 full articles and 50 abstracts were obtained from above mentioned databases. Thereafter different techniques and data were analyzed, categorized and compared with each other. Finally unpredictable, digressive and redundant data were discarded and the main collected points were arranged, summarized and divided into different sections.

## 3. Results

Resultant items and findings about classical and modern approaches used for viral hepatitis diagnosis were collected in different parts including immunological methods, molecular approaches and advanced biosensors. An analytical outline of each class of detection method presented below.

### 3.1. Immunoassay

Hepatitis virus derived antigens and antibodies are the main subjects for all immunoassay techniques. Antibodies as the detector elements play the central role in different formats of immunoassay methods. The most important and usual serological methods to detect hepatitis viruses include; enzyme immunoassay (EIA), radio-immunoassay (RIA) immuno-chromatographic assay (ICA), and immuno-chemiluminescence.

#### 3.1.1. Enzyme-Linked Immunosorbent Assay 

**Figure 1.**
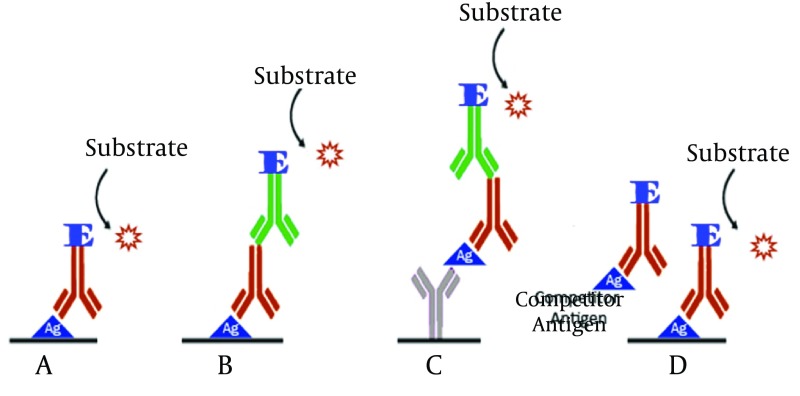
Based on the Layout Experiment and Antigen and Antibody Alignment, ELISA Can Be Classified Into Four Different Models A. Direct ELISA; B. Indirect ELISA; C. Sandwich ELISA; D. Competitive ELISA

Enzyme-linked immunosorbent assay (ELISA) accounts as a high sensitive method to detect many samples during a short time. The main component of ELISA is an enzyme conjugated antibody, the activity of which, is to produce a colored production (14). ELISA can accurately determine the presence of derived antigens or raised antibodies against hepatitis viruses (15). ELISA procedure is performed in four formats (Figure 1). ELISA kits are widely used to detect hepatitis viruses in clinical laboratories. In recent years, optimized ELISA methods have been developed to detect hepatitis in more accurate and sensitive ways through changing of influencing ELISA factors. In this regard, Kuo and colleagues in 2012 designed an ELISA technique to measure anti-HDV antibody. In this method a recombinant 127 amino acid HDAg, was used as the specific target. The sensitivity and specificity of their model were 97.3 and 100% respectively in comparison with RIA (16). Ansaldi and colleagues have also evaluated a model of ELISA for simultaneous detection of anti-HCV antibody and HCV core antigen (17).

#### 3.1.2. Radioimmunoassay

Radioimmunoassay (RIA) is the first generation of serological techniques and is a suitable tool to detect hepatitis viruses. In RIA a cold antigen (a non-radioactive one) competes with a hot antigen (an antigen conjugated with radioactive materials) for binding to antibodies (18). Therefore target concentration (non-conjugated target) and radiation have the inverse relation. Lander et al. are the pioneers of using modified RIA to detect hepatitis-associated antigen (HAA) followed by (19) countless studies on hepatitis viruses, performed on the basis of radioimmunoassay. Nowadays RIA is an extensively used technique to diagnose all types of hepatitis viruses in a commercial kit format.

#### 3.1.3. Immunochemiluminescent Assay

In 1976 the chemiluminogenic (CL) reagent was used for the first time as a non-isotopic label in an immunoassay system. CL labels are categorized into consumed and non-consumed labels, both employed for detection of viral hepatitis (20). Oxidation of CL reagents is associated with a measurable light emission (21). Immunochemiluminescent assay (CA) showed an equal and even higher level of sensitivity in comparison with RIA (22). Comparative studies on EIA and CA have confirmed that CA has equivalent sensitivity but relatively higher specificity, predictive value and fewer false-positive results in viral hepatitis diagnosis procedures. The high stability after conjugation, low consumption of reagents, high sensitivity and high safety are of other CA advantages. 

#### 3.1.4. Lateral Flow Immunoassay

**Figure 2.**
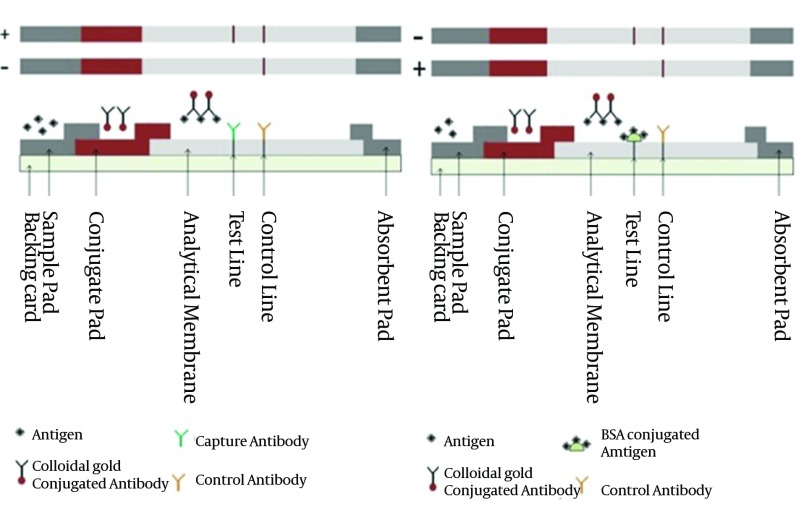
Tow Formats of Lateral Flow Immunoassay A) Sandwich format (antigen Sandwich) and testing results B) Competitive format and testing results. Positive (+) test and negative (-) test.

Lateral flow immunoassay (LFIA or LFA), due to its excellent advantages such as high sensitivity and specificity, ease of interpretation, simplicity of use and design and not requiring special instruments, has obtained extensive acceptance in clinical laboratories and being considered as a popular detection test (23). LFA works as a rapid test on the basis of fluid capillary movement through the sandwich (antigen or antibody sandwich) and commutative formats (Figure 2). LFA strip structure are composed of different overlapped layers include sample pad, conjugate pad, analytical membrane and absorbance pad. The liquid sample migrates through strip from sample pad to absorbance pad. Based on LFA format the appearing and disappearing of test lines determine the presence or absence of target in sample (24).

LFA is used for rapid detection of hepatitis viruses and human immunodeficiency virus (HIV). Since the first use of LFA for detection of hepatitis viruses, many modifications were done to improve its function in order to make the sensitivity and detection limits of some novel LFA comparable to ELISA (25, 26). Nowadays several commercial LFA kits with high sensitivity levels are available for detection of hepatitis viruses.

### 3.2. Molecular Based Detection

When serological techniques are not definitive, molecular assays are very helpful for the detection of all viral hepatitis infections, especially hepatitis B and C (10). Some molecular based detection methods like real-time PCR are able to quantify hepatitis viruses DNA or RNA copy numbers accurately. Nucleic acids tests (NAT) are performed in three formats including: non amplified, amplified and advanced methods among which amplified based methods are the most important and applicable for hepatitis virus detection (10). The invention of polymerase chain reaction (PCR) is owed to amplified nucleic acid techniques. PCR and its related techniques have created an incredible revolution in viral hepatitis diagnostic procedures. Hear the efficient systems for hepatitis viruses' molecular detection are discussed.

#### 3.2.1. Normal and Multiplex Polymerase Chain Reaction 

PCR standing for polymerase chain reaction can be utilized as a valuable implement to diagnose pathogenic agent through amplification of nucleic acids conserve region (DNA or RNA). PCR technique provides a condition for synthesis of million copies of interest nucleic acid segment in a short time. Multiplex PCR is a PCR procedure in which two or more different DNA target segments are amplified in a single tube. Detection power of PCR is as few as one to ten copy number of target. PCR and its related techniques have been widely used to diagnose both DNA and RNA hepatitis viruses. Several studies proved that PCR can detect HBV DNA even in samples which in, all serological markers are negative except for anti-HBc (hepatitis B virus core antibody) (3). In HBV acute and chronic infection forms, HBV DNA is detectable whereas all serological tests are negative (27). Multiplex PCR provides a situation in which several genotype and sub-genotypes of HBV can be detected simultaneously in a single tube (28).

#### 3.2.2. Nucleic Acid Sequence-Based Amplification and Transcription-Mediated Amplification

 Nucleic acid sequence base amplification (NASBA) and transcription mediated amplification (TMA) are of target amplification molecular techniques (29). Both techniques are designed to amplify and detect RNA and therefore, are very suitable to detect RNA hepatitis viruses such as HCV and HAV. NASBA is an isothermal RNA amplification reaction in which the synthesis of RNA is performed at the presence of a pair of primer and three enzymes including: reverse transcriptase (RT), RNase H and T7 RNA polymerase. The first NASBA detection assay for amplification of HCV RNA was described in 1994 by Sillekens et al. (30) TMA too is based on RNA amplification using both RNA and DNA as templates. RT and RNA polymerase play the enzymatic role in TMA procedure. NASBA method is more suitable for detection of HAV and HCV-RNA. Normal NASBA shows very poor amplification capabilities for HBV-DNA (31). On the other hand, the TMA technique can directly use both RNA and DNA as template, making it suitable for detection of HBV-DNA.

#### 3.2.3. Real-Time Polymerase Chain Reaction

Real-time PCR or quantitative PCR (qPCR) is a superior technology, able to diagnose and quantify hepatitis viruses based on nucleic acid amplification without post-PCR handling requirement (32). Nowadays several qPCR protocols are available to detect a number of clinically dangerous viruses, including hepatitis B and C viruses. Numerous advantages of real-time PCR including high sensitivity and specificity, high accuracy, broad dynamic range, capability to determine the quantity the viral load in specimens and many more have made this method the gold standard in hepatitis virus detection. Various fluorescent chemistries are used by qPCR to quantify DNA copy numbers. Fluorescent chemistries are generally divided into specific oligoprobes and non-specific compounds. For instance some dyes like SYBR green, non-specifically bind to double-stranded DNA while fluorescent resonance energy transfer (FRET) sequence-specific probes like dual hybridization probes specially bind to target sequences (33). In order to determine hepatitis genotypes and subtypes, qPCR is strongly able to characterize the genome of hepatitis viruses (34).

#### 3.2.4. Loop-Mediated Isothermal Amplification

Loop mediated isothermal amplification (LAMP) as a novel and strong method for nucleic acids amplification is considered an inexpensive and rapid tool for detecting hepatitis viruses (35). Since 2000, that Notomi and colleagues have developed LAMP technique, it has been widely used for diagnosis and quantification of viral pathogens. LAMP is a potent instrument for DNA amplification in detection of HBV (36, 37) which can also be used for detection of RNA hepatitis viruses (HAV, HCV and HEV) in reverse transcription way (38). Low time consumption, isothermal conditions and also no need for specific instruments are of this method's advantages (39). Nowadays different LAMP models are available as commercial diagnostic kits for several types of hepatitis viruses.

#### 3.2.5. Branched DNA

**Figure 3.**
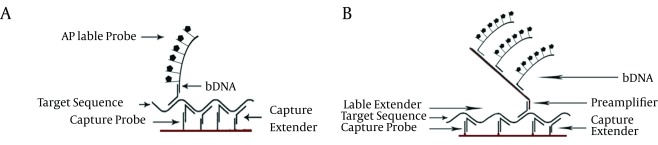
A Scheme for bDNA Assay A) First generation bDNA assay construction without preamplifier; B) second and third generation of bDNA assays. The preamplifier recruited in second and third generation bDNA assay increases the sensitivity of assay.

Branched DNA (bDNA) is a type of signal amplification technology, developed in early 90s for virus quantification. This method is initiated from hybridization of viral genomic materials with capture extenders and label extenders. Capture extenders are oligonucleotides designed to mediate the binding of targets to capture immobilized probes. Label extenders are designed to mediate binding of viral genome target to bDNA (40). Alkaline phosphatase conjugated oligonucleotide (AP-oligonucleotide) which is complementary to bDNA structure produces a signal by catalysis of alkaline phosphatase luminescent substrate (41) (Figure 3). The chemiluminescent emission resulting from AP reaction is monitored by a luminometer. Quantification of hepatitis viruses’ genome copy number is facilitated by bDNA assay through measuring the amplified signal.

### 3.3. Advanced Biosensors

New designed biosensors are accounted as very interesting and useful tools for detection of viral hepatitis infections. These are new label-free devices that can sense specific targets through bimolecular interactions such as enzyme–substrate reactions, receptor–ligand or antibody–antigen complexes (42). In general a biosensor is a combination of three parts;

1. biological detector elements like enzymes, immunological bioactive molecules, bio-receptors and probes nucleic acids,
2. transduction methods including mass-sensitive, electrical, electrochemical, optical and thermal transducers (43)
3. monitoring equipments (44).

Transducer elements or transduction methods are appropriate subjects for classification of biosensors (43). Among above mentioned transducers, thermal type is not a common applicable transducer to diagnose hepatitis viruses. Mass-sensitive transducers recognize small masses that bind to the surface of a specific miniaturized lever. This technology indebted to piezoelectric crystals such as quartz which is able to detect little changes in mass, through measuring the vibration frequency created by the electrical signals. Electrochemical detectors are another transducer to measure the variety of electrical signals caused by chemical reactions (45). Voltammetric and amperometric biosensors are more frequently used for detecting viral hepatitis infections than impedimetric biosensors (46). Optical transducer uses the optical signals as a detectable parameter. This transducer has the most sensitivity among all other transducers. Optical transducers are used to manufacture very sensitive detection methods like surface plasmon resonance (SPR) and optical based microcantilever, for viral hepatitis infection detection, (47). Detection of hepatitis viruses by described biosensors can be performed in a low-cost, reliable and rapid procedure.

#### 3.3.1. Surface Plasmon Resonance-Based Method

**Figure 4.**
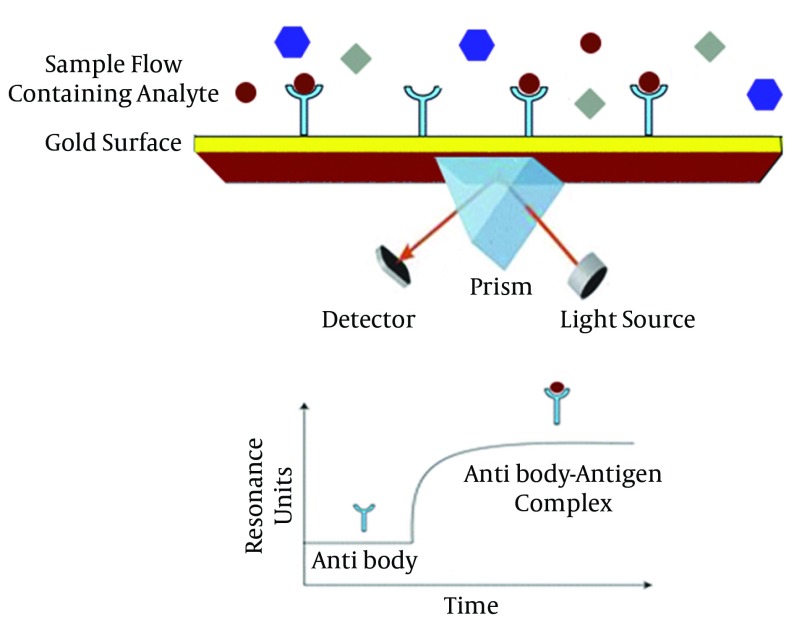
Schematic Representation of Surface Plasmon Resonance (SPR)

 Surface Plasmon Resonance (SPR) is considered as a significant instrumentation for analytical research, clinical diagnosis, in food industry, environmental pollution controlling and medical approaches (48). One SPR technique utilizes refractometric sensing devices using evanescent electromagnetic waves to analyze surface effects. It is a very flexible technology to be merged with various bio-recognition elements like antigens, antibodies, acid nucleic specific probes and aptamers. It is concluded that this method, is able to diagnose hepatitis viruses in a multi-pronged way. As a general overview, this techniques sense the changes in refractive index when the target molecules like HBV antigen bind to immobilized ligands (HBV antibody) on the thin surface. Each target binding affecting the resonant angle, is able to change the refractive index (49). Alterations in refractive index, without labeling requirement, can create a remarkable signal. Figure 4 illustrates a simple SPR mechanism. During recent years, many studies have been performed to improve and develop SPR-based methods for detection of viral hepatitis infections. Surface plasmon fluorescence spectroscopy (SPFS) (50) and surface plasmon resonance imaging (SPRI) are of the main SPR derived methods used for this aim.

#### 3.3.2. Piezoelectric Based Biosensor

Piezoelectric biosensors are devices that measure the mechanical pressure using the piezoelectric effect (conversion of mechanical stress to an electrical signal and vice versa). Piezoelectricity, discovered by Jacques and Curie in 1880 (51) is a trait of piezoelectric materials. Quartz crystal microbalance (QMC) is a piezoelectric based technology, used for detection of hepatitis B and C virus (52). QCM can detect small masses that bind to the surface of quarts piezoelectric surface. In a QCM detection method designed for hepatitis virus detection, a recognition element is immobilized on a coated quartz crystal electrode (52). After preparation of detector electrode, a soft flow of sample solution is used throughout. The interaction between target and recognition elements is monitored by measuring the resonant frequency of quartz crystals due to the mass changes on the electrode surface (52). Detection limitations of piezoelectric extremely depend on selection of the coating material and recognition element. Some of the advantages of piezoelectric-based detection devices are being label-free and direct monitoring of molecular interaction made which make it a suitable tool for viral hepatitis detection.

#### 3.3.3. Microcantilever Based Biosensors

Microcantilever as a mass sensitive device is able to sense different targets through detecting the shifts in cantilever deflection and vibration frequency. Similar to a miniaturized diving board microcantilever, is bended when a mass of the special target bounds (53). Microcantilever detection principle for hepatitis viruses is similar to piezoelectric based biosensor, mentioned above. In a brief description, hepatitis capturing nucleic acid probes or hepatitis specific immunological active elements (antigen or antibody) gets immobilized at the surface of microcantilever and later, carrier sample solution is added. Every hybridization or absorption operation on the surface of cantilever, leads to a detectable deflection (54). The deflecation of microcantilever is detected by several methods among which the piezoresistive and optical detection methods are the most applicable techniques (55). The sensitivity of optical detection methods is higher than piezoresistive methods (53). Some results obtained from microcantilever based biosensor, by using optical detector in the field of hepatitis virus detection, are comparable to some standard and sensitive detection methods like ELISA and chemiluminescence immunoassay (55).

#### 3.3.4. Electrochemical Biosensors

Electrochemical biosensors are accounted as the multifunctional devicesrecruiting electrochemical transducers (amperometric, voltammetric, impedimetric and condutometric) to detect some biological events like nucleic acids hybridization, enzymatic reactions and antigen-antibody complex formation and receptor-ligand bindings. Amperometric and voltammetric biosensors are widely used to detect hepatitis virus related genomic materialsm antibodies and antigens. Amperometic biosensors are more sensitive tools compared to voltammetric biosensors. In a typical amperometric biosensor, a biorecognition element such as enzyme, enzyme labeled antibody or antigen and a specific enzyme conjugated probe (or other electeroactive species), is employed to detect specific substrate, antigen, antibody and complementary sequences respectively. Multiple amperometric biosensors have been constructed to detect antigens, antibodies or genomic materials, derived from hepatitis viruses (46). Voltammetric transducers are another class of electrochemical biosensors working based on two elements including ion selective electrodes (ISE) used for converting the specific ion activity into electrical signals and ion sensitive field effect transistors (ISFET) used for determining the ion concentrations. In a developed voltammetric DNA biosensor for HBV diagnosis, a specific single strand HBV DNA (probe) was immobilized on a gold electrode. Gold electrode paste probe was placed in contact with PCR amplified HBV DNA fragments and hybridization reaction was investigated by osmium bipyridine as an electroactive indicator. Specific accumulation of osmium bipyridine on the gold electrode surface due to DNA hybridization, extremely enhanced the current peak, compared to those obtained for probe-modified electrode. The results of this study also show a good sensitivity for determining even one-base mismatch, during nucleic acid hybridization, which makes it a suitable device for hepatitis virus genotyping. 

#### 3.3.5. Apta-sensors

**Figure 5.**
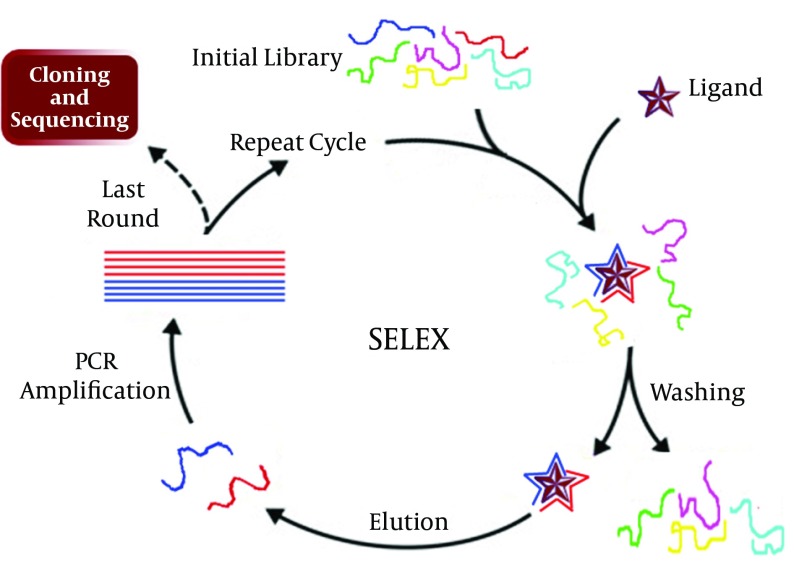
Systematic Evaluation of Ligand by Exponential Enrichment (SELEX) for selecting a suitable aptamer. A rate equal between 10 to 15 Rounds is essential to reach a proper aptamer

Aptamers are defined as RNA made biomolecules, single strand DNA or peptide molecules with the ability to bind specifically to their target molecules. A specific aptamer is selected among 1015 different sequences using a reiterative screening procedure called systematic evaluation of ligand by exponential enrichment (SELEX) (Figure 5). Research results have shown Research results have shown that the detection limits of aptamers to their targets have a range of picomolar (pM) to micromolar (μM). Nowadays aprtamers known as apta-sensors, are widely used for construction of sensitive and specific biosensors, for diagnosis of widespread ranges of clinical diseases. Aptamers are good alternative recognizer elements, used for detection of hepatitis viruses in a wide range of methods like nanomechanical microcantilever, enzyme-linked oligonucleotide assay (ELONA) (56), aptamer-linked immobilized sorbent assay (ALISA) and lateral flow assay (LFA), and other biosensors (56). Several apta-sensors have been developed for detection of hepatitis viruses, like the highly sensitive ELONA developed for detection of HCV core antigen. In this technology an aptamer was immobilized on microtitration plates and an enzyme conjugated monoclonal antibody was added after HCV core antigen capturing. The detection procedure was completed by addition of the enzyme substrate. In another instance, RNA aptamers were used as a capturing element by Hwang et al. developing a nanomechanical microcantilever for sensitive detection of HCV helicase. Their sensor was able of detecting targets at concentrations as low as 100 pg/mL HCV helicase (57).

## 4. Conclusions

Viral hepatitis is considered a dangerous public health problem worldwide. Diagnosis and treatment are the main goals to inhibit the spread of hepatitis viruses. Exact diagnosis of hepatitis virus types and infection stage for an effective treatment need a relatively broad knowledge about viral hepatitis infections. Each detection technique has its special advantages and limitations. EIAs are the most important serological assays used for hepatitis viruses’ detection. EIAs procedures are simple and convenient to set up and having nano-gram or sensitivity to low levels of target presence, few reagents requirement, quantitative and qualitative testing and capability of being automated are some of the advantages of EIAs. On the other hand they can be time-consuming and expensive devices. Rapid detection of viral hepatitis, soon after infection, is an urgent requirement to treat and prevent infection transmission. Development of molecular methods for diagnosis of viral genomic materials has revolutionized the detection procedure in clinical laboratories. Some of these techniques have been introduced as common laboratory tests but some others such as real-time PCR are applied as gold-standard and reference settings. The major advantages of molecular methods are having higher specificity and sensitivity and larger dynamic range of action compared to other diagnostic methods like serological assays. Regarding nucleic acid tests, requiring special instruments and post handling in some molecular tests and inability to indicate the pathogen viability are the main limitations. Biosensors have been described for label free detection of hepatitis viruses. Most of biosensors are based on the combination of existing molecular and immunological techniques coupled with optical, electrochemical, mass-sensitive and electrical sensing modules. The main advantages of these detection systems are offering a quantitative test for detection in cases with about 100 copies of hepatitis virus, in addition to automation, multiplexing analysis and throughput. It seems further studies are necessary to completely uniform the methods that can be established as universal recommendations for detection of hepatitis viruses.
